# The Role of Cytokines and Chemokines as Biomarkers of Disease Activity in Idiopathic Nephrotic Syndrome in Children

**DOI:** 10.3390/cimb47020077

**Published:** 2025-01-25

**Authors:** Matjaž Kopač, Aleš Jerin, Agnese Petrera, Joško Osredkar

**Affiliations:** 1 Department of Nephrology, Division of Paediatrics, University Medical Centre Ljubljana, Bohoričeva 20, 1525 Ljubljana, Slovenia; 2 Faculty of Medicine, University of Ljubljana, Vrazov trg 2, 1000 Ljubljana, Slovenia; 3 Institute of Clinical Chemistry and Biochemistry, University Medical Centre Ljubljana, Zaloška cesta 2, 1525 Ljubljana, Slovenia; 4 Faculty of Pharmacy, University of Ljubljana, Aškerčeva 7, 1000 Ljubljana, Slovenia; 5 Metabolomics and Proteomics Core, Helmholtz Zentrum München—German Research Center for Environmental Health, 85764 Neuherberg, Germany

**Keywords:** cytokines, chemokines, idiopathic nephrotic syndrome, children

## Abstract

(1) This study investigates the association of plasma concentrations of various cytokines and chemokines with the disease activity of idiopathic nephrotic syndrome (INS) in children in Slovenia. (2) In a prospective single-center study lasting 18 months, we took sequential plasma samples from children with INS at disease onset or relapse (prior to corticosteroid (CS) therapy), at remission, and after discontinuation of CS therapy. The Olink^®^Target 48 Cytokine Panel was applied to analyze 45 analytes in the plasma samples, adhering to the manufacturer’s protocol. We performed a statistical analysis with a paired samples analysis with a *t*-test as well as with a non-parametric Wilcoxon correction test. (3) We included 18 pediatric patients with INS in the study. We demonstrated statistically significant differences in the concentrations of CSF1, IL4, FLT3LG, CCL19, and MMP12 in the patients at disease onset or relapse compared to those in remission, differences in the concentrations of CSF1 and IL17F in the patients at disease onset or relapse compared to those in remission after CS treatment, and differences in the concentrations of CCL19, MMP12, and CCL13 in the patients in remission compared to those in remission after CS treatment. (4) The findings support potential roles of certain cytokines and chemokines, especially CSF1, CCL19, and MMP12, in influencing the disease activity of INS.

## 1. Introduction

### 1.1. Overview of the Studied Clinical Problem

Nephrotic syndrome is a kidney disorder characterized by significant proteinuria, hypoalbuminemia, hyperlipidemia, and edema. The disease can be classified into idiopathic nephrotic syndrome (INS) and secondary nephrotic syndrome. INS is often immune-mediated and lacks a clear underlying cause, while secondary nephrotic syndrome is associated with systemic diseases like systemic lupus erythematosus (SLE) or infections. Inflammation plays a crucial role in the pathophysiology of nephrotic syndrome and also in other kidney diseases. Several cytokines and chemokines are implicated in driving the inflammatory response, altering glomerular filtration, and promoting podocyte damage. INS is the most common glomerular disease in children, with an incidence of 1.15–16.9/100,000 children depending on ethnicity and region [1,2,3]. This disease was associated with a high mortality rate (about 40%) as a consequence of acute kidney injury (AKI), chronic kidney disease (CKD), systemic infections, and thromboembolic complications until the discovery of corticosteroids (CS) as an effective therapy in the 1950s. Most affected children (about 85%) show complete remission of proteinuria within four to six weeks with daily CS treatment, but 70–80% will suffer from at least one relapse during follow-up. In addition, about 50% of these patients have frequent relapses or are steroid-dependent. Relapse is defined, according to the International Pediatric Nephrology Association (IPNA) clinical practice recommendations, as the presence of nephrotic-range proteinuria, which is ≥3 + (≥300 mg/dL) on a urine dipstick, a urinary protein/creatinine ratio (UPCR) ≥ 200 mg/mmol (≥2 mg/mg) on a spot urine sample on three consecutive days, or proteinuria above 1 g/m^2^ of the body surface area in a 24 h urine collection, with or without the reappearance of edema in a child who had previously achieved complete remission [1,2,4]. The disease can resolve spontaneously following puberty, but 10–30% continue to have relapses into young adulthood. Kidney biopsy is not routinely indicated in pediatric patients because of its limited prognostic or clinical utility. However, when this is conducted, the most common diagnosis is minimal change disease (MCD), characterized by podocyte foot process effacement, while focal-segmental glomerulosclerosis (FSGS) is much less common. The treatment of INS with relapses is difficult because long-term CS use is associated with steroid side effects and decreased quality of life (QOL). Several steroid-sparing agents are available, but they can cause serious side effects as well [1].

Cytokines and chemokines play crucial roles in the development and progression of INS, other kidney diseases, and immune-mediated disorders in children. These molecules mediate inflammatory responses, recruit immune cells to the site of injury, and contribute to tissue damage or repair.

The aim of this research was to study potential associations of various cytokines and chemokines with disease activity in order to better understand the etiopathogenesis of INS, which is still largely unknown. These findings could help in developing more personalized therapies for INS, which have been non-selective for many years, consisting of CS, with the addition of other non-selective immunosuppressive drugs when necessary.

Therapies targeting specific cytokines, such as IL-6 inhibitors (e.g., tocilizumab) and TNF-α blockers (e.g., infliximab), are already being explored for their potential to reduce inflammation and proteinuria in INS. These targeted therapies offer promising alternatives to traditional immunosuppressive treatments [5].

### 1.2. Proinflammatory and Anti-Inflammatory Cytokines and Chemokines in INS

Cytokines and chemokines are key regulators of the immune system and play a central role in the pathogenesis of various kidney diseases, including INS, as well as other immune-mediated diseases. Elevated levels of proinflammatory cytokines such as IL-6, IL-18, IL-1β, and TNF-α have been proposed to be associated with disease activity in INS. These cytokines contribute to glomerular inflammation and increased permeability, leading to proteinuria. High levels of IL-13 have been specifically linked to relapses [6].

**Figure 1:**
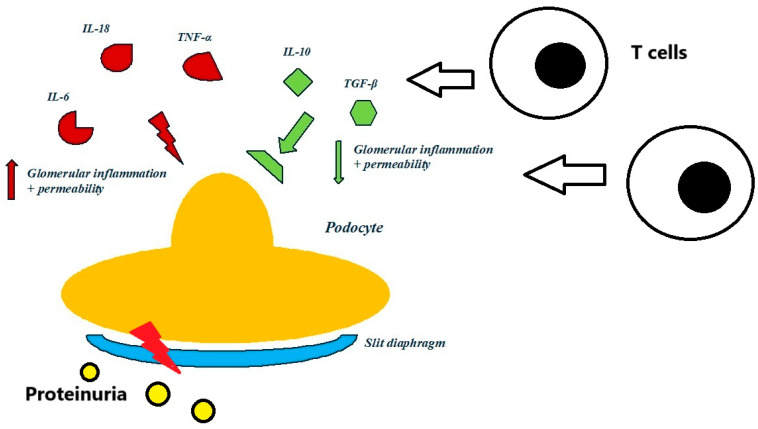
Schematic representation of the role of some proinflammatory (in red colour) and anti-inflammatory (in green colour) cytokines and chemokines in etiology of idiopathic nephrotic syndrome [6,7].

Anti-inflammatory cytokines like IL-10 and transforming growth factor-beta (TGF-β) play protective roles by modulating immune responses and reducing inflammation. A delicate balance between proinflammatory and anti-inflammatory cytokines is crucial for disease progression and remission [7]. Figure 1 schematically presents the roles of some proinflammatory and anti-inflammatory cytokines and chemokines in the etiology of INS [6,7].

## 2. Materials and Methods

### 2.1. Clinical Study Design

In this prospective single-center study, with 18 months duration, taking place at Department of Nephrology, Division of Paediatrics, University Medical Centre Ljubljana in Slovenia, we took three sequential heparin-plasma samples (whenever feasible) from children with INS in the following manner:

- at disease onset or relapse confirmation, prior to initiation of CS therapy, in order to abate its potential influence on results of laboratory investigations in this phase of the disease;
- at remission achievement with CS therapy, after which this therapy was gradually tapered over about eight weeks until discontinuation;
- after discontinuation of CS therapy in order to abate its potential influence on results of laboratory investigations in this phase of the disease.

We collected three samples per patient in most cases; however, we were able to obtain only one or two samples per patient in some instances. This was due to various reasons in real-life clinical setting, such as relapse soon after discontinuation of CS therapy, stable disease in long-term remission, or failure to attend or postponement of a scheduled patient’s follow-up visit.

Study design, using Olink^®^Target 48 Cytokine Panel, was longitudinal with multiple time points per patient, as described above. In this way, we attempted to follow the longitudinal cytokine changes over time, during the treatment period and beyond. Randomization across the plates and intensity normalization could thus be achieved as patients’ time points were consistent.

We obtained written informed consent from all parents of the included pediatric patients and themselves (if above 14 years old) prior to sample collection. The study was approved by Slovenian National Committee for Medical Ethics, number 0120-501/2022/3.

### 2.2. Analytical Method

Laboratory analysis was conducted at Metabolomics and Proteomics Core Facility (MPC), Helmholtz Zentrum München—German Research Center for Environmental Health. Olink^®^Target 48 Cytokine Panel was applied to analyze 45 analytes in plasma samples, adhering to the manufacturer’s protocol (Olink^®^—Olink Proteomics AB, Uppsala, Sweden, link to the product: https://olink.com/products/olink-target-48, accessed on 20 December 2024). We used 96-well PCR plate full skirt (Thermo Fisher Scientific #AB0800, Waltham, MA, USA) as shipment plate. This method is based on the Proximity Extension Assay (PEA) technology, as extensively detailed by Assarsson et al. [8]. Briefly, for each protein assay, a matched pair of antibodies linked to unique oligonucleotides binds to the respective protein target. When placed in close proximity, the stretch of nucleotides hybridizes and the annealing product is then amplified by PCR and detected in multiplexed fashion in a high-throughput fluidic chip system named Olink Signature Q100 instrument. Data analysis and quality control were conducted using the Olink NPX Signature Software version: 1.15.0. To ensure accuracy, each run included internal controls and calibrators. Data normalization was performed using an internal extension control and calibrators, thereby effectively minimizing any intra-run variability. The conversion of NPX-values to protein concentrations in standard units (pg/mL) was performed by the Olink NPX Signature software using a robust 4-parameter logistic (4-Pl) fit model. Assay validation parameters, including limits of detection and intra- and inter-assay precision data, are available at www.olink.com.

Identification of true biological differences between study groups was facilitated by reducing technical variability to the fullest extent possible. This included using the same collection procedure for each sample and maintaining even storage conditions. The same storage conditions have been applied to all samples regarding temperature, storage time, freeze–thaw cycles, collection site, and collection tube. However, the biological variables were not equally distributed across study groups.

Randomization of samples was conducted in order to empower our study and minimize the risk of introducing any bias that could confound downstream analyses. If randomization had not been performed, the normalization might have removed true biological variation that otherwise could have been identified. For this reason, variables present in our cohort were balanced across and within plates. Samples in a longitudinal study that were collected from the same individual at different time points were all included on the same plate to further reduce variations in the data. In addition, the longitudinal samples were not placed near each other, and the time points of the same patient were distributed over the plate. 

### 2.3. Statistical Method

We performed statistical analysis, including paired samples analysis with *t*-test as well as non-parametric Wilcoxon correction test, because the distribution of sample was not normal. Statistical significance of concentration differences regarding various cytokines and chemokines in plasma samples was presented as *p*-value, with value less than 0.05 considered statistically significant.

### 2.4. Biologic and Demographic Data of Included Patients

Eighteen patients with INS, aged between 3 and 19 years, were included in the study, sixteen boys and two girls. Thirteen of them had kidney biopsy and all of them had MCD. Four of them were new cases at disease onset, nine had relapse, and five of the included children were in stable remission. Four of the included children were receiving vitamin D, two of them therapy with CS, and six of them other immunosuppressive drugs (three of them mycophenolate-mofetil (MMF), one cyclosporine, and two tacrolimus) at time of relapse. 

## 3. Results

The results of the laboratory analysis of the studied cytokines and chemokines in those children with INS that demonstrated statistically significant differences are presented in Table 1. However, all the other results of laboratory investigations, including concentrations, average values, minimal and maximal values of studied cytokines and chemokines, as well as measures of statistical significance (expressed as *p*-values) are available in the Supplementary Materials (tables and graphs).

The results in Table 1 show statistically significantly higher concentrations of the following cytokines and chemokines in the plasma samples of patients at disease onset or relapse compared to those in remission achievement with CS treatment: CSF1 (macrophage colony-stimulating factor 1), IL4 (anti-inflammatory cytokine interleukin-4, enhancing B-cell differentiation and regulating immune response, increased in allergic reactions and inflammations), CCL19 (proinflammatory chemokine C-C motif chemokine 19, attracting immune cells, especially dendritic cells, into lymph nodes, increased in inflammatory processes and infections), MMP12 (macrophage metalloelastase 12), and FLT3LG (Fms-related tyrosine kinase 3 ligand, anti-inflammatory growth factor, active during immune system activation).

The results in Table 1 also indicate statistically significant differences in the concentrations of the following cytokines and chemokines in the plasma samples of patients at disease onset or relapse compared to those in remission after termination of treatment with CS: CSF1 (described above) and IL17F (proinflammatory cytokine interleukin-17F, enhancing inflammation and defense against infections, increased in autoimmune disease and infections).

In addition, the results in Table 1 show statistically significantly lower concentrations of the following cytokines and chemokines in the plasma samples at the time of remission achievement with CS treatment compared to those in remission after termination of treatment with CS: CCL19, MMP12 (both described above), and CCL13 (proinflammatory chemokine C-C motif chemokine 13, attracting monocytes and lymphocytes to sites of inflammation, increased in chronic inflammatory conditions).

## 4. Discussion

Cytokines and chemokines are key regulators of the immune system and play a central role in the pathogenesis of various kidney diseases, including INS, as well as other immune-mediated diseases. These molecules are responsible for mediating and orchestrating immune responses, inflammation, and tissue remodeling. In children, immune-mediated diseases are of particular concern due to their impact on the developing body, making it critical to understand the role of specific cytokines and chemokines in these conditions. This research explores the involvement of various cytokines and chemokines in the pathogenesis of INS in children.

Elevated levels of IL-18, a proinflammatory cytokine involved in the regulation of immune responses, have been detected in children during relapses of INS [9]. Reduced levels of HGF, a growth factor with anti-apoptotic and pro-survival effects on podocytes, have been associated with the progression of nephrotic syndrome [10]. CCL2 plays a central role in recruiting monocytes and macrophages to sites of inflammation and has been associated with the development of glomerular and tubular injury in kidney diseases [11]. Pathway activation of TNF, a proinflammatory cytokine that contributes to podocyte injury, glomerular inflammation, and apoptosis of podocytes, has been described to cause proteinuria and poor outcomes in some patients with INS [12,13], especially in those with steroid-resistant nephrotic syndrome (SRNS) [14]. Several other cytokines and chemokines, such as C-X-C motif chemokine 10 (CXCL10), IL-17A, IL-6, interleukin-1 beta (IL-1β), and granulocyte colony-stimulating factor (G-CSF), have been, according to various studies, proposed to play a pathogenic role in children with INS and other glomerular diseases [15,16,17,18,19]. Likewise, the plasma concentrations of proinflammatory cytokines IL-6 and IL-8 have been reported to be significantly higher in children with SSNS compared to SRNS and were proposed as biomarkers for the prediction of CS response [20]. Vascular endothelial growth factor A (VEGF-A), a proinflammatory growth factor and regulator of angiogenesis and vascular permeability, has been reported to have increased expression in diabetic nephropathy, associated with increased glomerular permeability and proteinuria [21]. However, the pathogenic role of either of the described molecules in the disease activity of INS has not been confirmed in our study.

CSF1, a proinflammatory growth factor, stimulating macrophages to migrate to inflammatory processes and chronic inflammation, has been confirmed to be associated with disease activity in our study, especially in the patients at disease onset or relapse compared to those in remission achievement and those in remission after termination of treatment with CS, suggesting its potential role in disease activity, which seems to be present even in the absence of CS treatment. CSF1 controls the survival, proliferation, and differentiation of macrophages, which serve as scavengers, with a role in the innate and acquired immune response. Macrophages have many other roles during development and tissue homeostasis because of their plasticity. There is evidence that CSF-1 plays an important trophic role in postnatal organ growth and kidney repair. CSF-1 seems to promote postnatal renal repair in mice after ischemia–reperfusion injury. In addition, CSF-1 treatment quickly accelerated renal repair with tubular epithelial cell replacement, attenuation of interstitial fibrosis, and functional recovery. These results show that CSF-1 is important in kidney growth, repair, and resolution of inflammatory injury. Targeting CSF1 could, therefore, potentially reduce macrophage-mediated inflammation and glomerular injury [22].

The role of MMP12 (macrophage metalloelastase), a proinflammatory enzyme that degrades matrix proteins such as elastin, which leads to tissue remodeling, has been found to be elevated in chronic lung diseases and inflammatory processes. However, its functions in kidney diseases have not yet been completely discovered. A published study researched the mechanism of kidney fibrosis, exploring the expression and localization of MMP-12 and associated regulatory molecules in the kidneys of experimentally induced glomerulonephritis in mice. Significant expression of *MMP-12* mRNA and its protein in animal kidney was confirmed, especially in podocytes, which lead to progression to nephrotic syndrome. These findings imply the involvement of the expression of MMP-12 in the progression of nephrotic syndrome in animal models. Inhibition of MMP12, therefore, might protect against podocyte injury by reducing extracellular matrix degradation [23]. These findings have been confirmed in our study because we have demonstrated statistically significantly higher concentrations of MMP12 in the plasma samples of the patients at disease onset or relapse than in those in remission achievement with CS treatment as well as lower concentrations in the plasma samples of the patients at remission with CS treatment compared to those in remission after termination of treatment with CS. This might suggest an important role of MMP12 regarding disease activity, with the potential to be a biomarker of disease activity.

RNA sequencing in a recent study revealed that IL-4 can induce changes in the expression of genes in human podocytes. Their clinical significance is not yet completely understood, but they may be involved in the induction of INS as their expression levels were found to be reversed by CS therapy, suggesting that molecular players may be involved in the induction of IL-4-mediated podocyte changes in an in vitro setting [24]. Another study revealed increased levels of IL-4 in relapse of nephrotic syndrome and slightly lower in disease remission. In addition, a positive correlation was noticed between IL-4 and 24 h proteinuria. It was concluded that the increased IL-4 production of T cells might account for the elevated serum IgE level and that IL-4 may play a pathogenetic role in INS [25]. These findings are in concordance with the results of our study, where we found statistically significantly higher concentrations of IL-4 in the plasma samples at disease onset or relapse compared to those in remission achievement with CS treatment.

Our study also revealed statistically significantly lower concentrations of IL-17F in the plasma samples at disease onset or relapse compared to those in remission after termination of CS treatment. However, treatment with CS might affect their concentrations. Likewise, a study on an animal model showed that IL-17F induced expression of neutrophil-attracting chemokines CXCL1 and CXCL5 in kidney cells, leading to the development of renal tissue injury, which could be important for the development of anti-IL-17 cytokine therapies in T_H_17-mediated human autoimmune diseases [26].

Other substances, proinflammatory chemokines CCL19 and CCL13, had significantly lower concentrations in the plasma samples of the patients at the time of remission achievement with CS treatment than those in remission after termination of CS treatment, suggesting their potential role as biomarkers of disease activity, obviously influenced by CS treatment. In addition, the CCL19 concentrations were significantly higher in those plasma samples at disease onset or relapse compared to those in remission with CS treatment. This is in agreement with a recent study that identified *CCL19* as a potential critical gene (and C-C motif chemokine 19 as its protein product) and immune-related biomarker of diabetic nephropathy as the upregulated level of CCL19 was confirmed in other independent datasets as well as in in vitro experiments with high glucose [27]. It is also in agreement with another study that evaluated the gene expression profiling of peripheral blood mononuclear cells from patients with MCD during the relapse and remission phases. Its results showed significantly increased mRNA expression of *CCL13* in all the included patients with relapse compared to those in remission, patients with membranous nephropathy, and healthy controls. The *CCL13* mRNA expressions in peripheral blood mononuclear cells, therefore, seem to be upregulated specifically in patients with MCD during the active disease phase, such as relapse [28].

In addition, our study revealed statistically significantly higher concentrations of FLT3LG in the plasma samples at disease onset or relapse than in those in remission achievement with CS treatment, suggesting its role as a potential biomarker of INS disease activity. Its role in INS or kidney diseases has not yet been reported. However, FLT3LG administration to laboratory animals improved the immune status and alleviated the organ damage in mice with multiorgan dysfunction syndrome, although the average number of CD4+ T lymphocytes in the treated group was not significantly different to the average number of CD4+ T lymphocytes in the healthy control group [29].

The potential clinical implications of these findings include that there are several emerging targets among various cytokines and chemokines, evaluated in our study as well, against which agents have been developed. Further research is necessary to expand this knowledge in order to better understand the etiopathogenesis of INS. These findings could, as a consequence, help to develop more personalized and selective therapies for INS. Therapies targeting specific cytokines, such as IL-6 inhibitors (e.g., tocilizumab), TNF-α blockers (e.g., infliximab), and CCX140-B (an antagonist of the chemokine receptor CCR2), have already been explored for their potential to reduce inflammation and proteinuria in INS. These targeted therapies offer promising alternatives to traditional immunosuppressive treatments [5,30]. Understanding the role of specific cytokines and chemokines, evaluated in our study as well, offers new opportunities for targeted therapies that can modulate the immune response, reduce inflammation, and preserve kidney function. However, the relatively small sample size is a limitation of this study. In addition, it included only patients with SSNS because no new patients with SRNS were present at our department during the study enrollment period. Otherwise, the results might have been different.

## 5. Conclusions

Our findings support the potential role of certain cytokines and chemokines, especially CSF1, CCL19, and MMP12, in influencing the disease activity of INS that could, therefore, serve as potential biomarkers of disease activity. Future research in larger cohorts of patients, including patients with SRNS, should be conducted to confirm these results as well as to focus on developing cytokine-targeted therapies to improve outcomes for children with INS and other immune-mediated kidney diseases.

## Figures and Tables

**Table 1:** Statistically significant concentration differences in cytokines and chemokines in plasma samples of patients at disease onset or relapse (group 1—G1) compared to those in remission achieved with corticosteroid (CS) treatment (group 2—G2) and to those in remission after conclusion of CS treatment (group 3—G3). All results of laboratory measurements are presented in pg/µL (except for IL-4—units presented in pg/mL due to better visualization). N—number of paired samples.

Cytokine/Chemokine	G1 Average	G1 [Min, Max]	G2 Average	G2 [Min,Max]	*p*-Value (G1 vs. G2)	*p*-Value (Wilcoxon Test)	N
CSF1	17,914.68	[13,500.14, 22,343.19]	13484.45	[9643.39, 16,363.25]	0.001	0.002	13
MMP12	28,607.54	[6436.08, 58,715.05]	15,874.35	[3696.65, 43,664.75]	0.036	0.033	13
FLT3LG	8445.84	[2788.73, 18,855.89]	5741.94	[1951.46, 9135.27]	0.050	0.040	13
IL4	0.0392	[0.008, 0.1632]	0.0102	[0.0072, 0.0175]	0.110	0.042	11
CCL19	10,939.78	[2311.75, 31,713.69]	6144.62	[912.61, 13,383.07]	0.042	0.068	13
	G1 average	G1 [min, max]	G3 average	G3 [min,max]	*p*-value (G1 vs. G3)	*p*-value (Wilcoxon test)	N
CSF1	18,456.84	[13,500.15, 22,343.19]	13,776.12	[11,727.92, 15,216.58]	0.003	0.016	8
IL17F	21.06	[0.00034, 168.49]	85.12	[0.000196, 209.44]	0.068	0.039	8
	G2 average	G2 [min, max]	G3 average	G3 [min,max]	*p*-value (G2 vs. G3)	*p*-value (Wilcoxon test)	N
CCL19	7743.25	[2344.13, 13,383.07]	17,541.20	[8872.62, 29,817.32]	0.015	0.016	8
MMP12	18,486.89	[3696.66, 43,664.75]	42,074.98	[15,489.89, 74,369.29]	0.032	0.039	8
CCL13	16,545.44	[9981.48, 26,466.91]	32,143.81	[7983.75, 54,187.33]	0.041	0.054	8

## Data Availability

The data that support the findings of this study are available from the study’s principal investigator, M.K., upon reasonable request.

## Supplementary Materials

The following supporting information can be downloaded at: https://www.mdpi.com/article/10.3390/cimb47020077/s1.

## Abbreviations

The following abbreviations are used in this manuscript:

IL18	Interleukin-18
HGF	Hepatocyte growth factor
CCL19	C-C motif chemokine 19
CCL2	C-C motif chemokine 2
MMP12	Macrophage metalloelastase
LTA	Lymphotoxin-alpha
FLT3LG	Fms-related tyrosine kinase 3 ligand
TNF	Tumor necrosis factor
IL17A	Interleukin-17A
IL2	Interleukin-2
IL17F	Interleukin-17F
CSF3	Granulocyte colony-stimulating factor
IL1B	Interleukin-1 beta
OLR1	Oxidized low-density lipoprotein receptor 1
TNFSF12	Tumor necrosis factor ligand superfamily member 12
CXCL10	C-X-C motif chemokine 10
VEGFA	Vascular endothelial growth factor A
IL33	Interleukin-33
TSLP	Thymic stromal lymphopoietin
IFNG	Interferon gamma
CCL4	C-C motif chemokine 4
TGFA	Protransforming growth factor alpha
IL13	Interleukin-13
CXCL8	Interleukin-8
CCL8	C-C motif chemokine 8
IL6	Interleukin-6
CCL13	C-C motif chemokine 13
CSF2	Granulocyte–macrophage colony-stimulating factor
CCL7	C-C motif chemokine 7
IL4	Interleukin-4
TNFSF10	Tumor necrosis factor ligand superfamily member 10
OSM	Oncostatin-M
MMP1	Interstitial collagenase
EGF	Pro-epidermal growth factor
IL7	Interleukin-7
IL15	Interleukin-15
CSF1	Macrophage colony-stimulating factor 1
CXCL9	C-X-C motif chemokine 9
CXCL11	C-X-C motif chemokine 11
IL17C	Interleukin-17C
CXCL12	Stromal cell-derived factor 1
CCL11	Eotaxin
IL10	Interleukin-10
CCL3	C-C motif chemokine 3
EBI3_IL27	Interleukin-27

## References

[1] Trautmann A., Boyer O., Hodson E., Bagga A., Gipson D.S., Samuel S., Wetzels J., Alhasan K., Banerjee S., Bhimma R. (2023). International Pediatric Nephrology Association. IPNA clinical practice recommendations for the diagnosis and management of children with steroid-sensitive nephrotic syndrome. Pediatr. Nephrol..

[2] Noone D.G., Iijima K., Parekh R. (2018). Idiopathic nephrotic syndrome in children. Lancet.

[3] Tullus K., Webb H., Bagga A. (2018). Management of steroid resistant nephrotic syndrome in children and adolescents. Lancet Child. Adolesc. Health.

[4] Carter S.A., Mistry S., Fitzpatrick J., Banh T., Hebert D., Langlois V., Pearl R.J., Chanchlani R., Licht C.P., Radhakrishnan S. (2020). Prediction of short- and long-term outcomes in childhood nephrotic syndrome. Kidney Int. Rep..

[5] Furutera N., Fukunaga N., Okita J., Suzuki T., Suenaga Y., Oyama Y., Aoki K., Fukuda A., Nakata T., Uesugi N. (2021). Two cases of idiopathic multicentric Castleman disease with nephrotic syndrome treated with tocilizumab. CEN Case Rep..

[6] Kalluri R. (2006). Proteinuria with and without renal glomerular podocyte effacement. J. Am. Soc. Nephrol..

[7] Yap H.K., Cheung W., Murugasu B., Sim S.K., Seah C.-C., Jordan S.C. (1999). Th1 and Th2 cytokine mRNA profiles in childhood nephrotic syndrome. J. Am. Soc. Nephrol..

[8] Assarsson E., Lundberg M., Holmquist G., Björkesten J., Thorsen S.B., Ekman D., Eriksson A., Dickens E.R., Ohlsson S., Edfeldt G. (2014). Homogenous 96-plex PEA immunoassay exhibiting high sensitivity, specificity, and excellent scalability. PLoS ONE.

[9] Zhou J., Shi F., Xun W. (2018). Leptin, hs-CRP, IL-18 and urinary protein before and after treatment of children with nephrotic syndrome. Exp. Ther. Med..

[10] Matsumoto K., Nakamura T. (2001). Hepatocyte growth factor: Renotropic role and potential therapeutics for renal diseases. Kidney Int..

[11] Souto M.F., Teixeira A.L., Russo R.C., Penido M.G., Silveira K.D., Teixeira M.M., Simões E. (2008). Immune mediators in idiopathic nephrotic syndrome: Evidence for a relation between interleukin 8 and proteinuria. Pediatr. Res..

[12] Jiang Y., Glasstetter L.M., Lerman A., Lerman L.O. (2023). TSG-6 (Tumor Necrosis Factor-α-Stimulated Gene/Protein-6): An Emerging Remedy for Renal Inflammation. Hypertension.

[13] Mariani L.H., Eddy S., AlAkwaa F.M., McCown P.J., Harder J.L., Nair V., Eichinger F., Martini S., Ademola A.D., Boima V. (2023). Precision nephrology identified tumor necrosis factor activation variability in minimal change disease and focal segmental glomerulosclerosis. Kidney Int..

[14] Weissbach A., Garty B.Z., Lagovsky I., Krause I., Davidovits M. (2017). Serum Tumor Necrosis Factor-Alpha Levels in Children with Nephrotic Syndrome: A Pilot Study. Isr. Med. Assoc. J..

[15] Cortvrindt C., Speeckaert R., Moerman A., Delanghe J.R., Speeckaert M.M. (2017). The role of interleukin-17A in the pathogenesis of kidney diseases. Pathology.

[16] Gao J., Wu L., Wang S., Chen X. (2020). Role of Chemokine (C-X-C Motif) Ligand 10 (CXCL10) in Renal Diseases. Mediators Inflamm..

[17] Jia W., Dou W., Zeng H., Wang Q., Shi P., Liu J., Liu Z., Zhang J., Zhang J. (2024). Diagnostic value of serum CRP, PCT and IL-6 in children with nephrotic syndrome complicated by infection: A single center retrospective study. Pediatr. Res..

[18] Afsar B., Covic A., Ortiz A., Afsar R.E., Kanbay M. (2018). The Future of IL-1 Targeting in Kidney Disease. Drugs.

[19] Yan J.J., Ryu J.H., Piao H., Hwang J.H., Han D., Lee S.K., Jang J.Y., Lee J., Koo T.Y., Yang J. (2020). Granulocyte Colony-Stimulating Factor Attenuates Renal Ischemia-Reperfusion Injury by Inducing Myeloid-Derived Suppressor Cells. J. Am. Soc. Nephrol..

[20] Nickavar A., Valavi E., Safaeian B., Amoori P., Moosavian M. (2020). Predictive Value of Serum Interleukins in Children with Idiopathic Nephrotic Syndrome. Iran. J. Allergy Asthma Immunol..

[21] Estrada C.C., Maldonado A., Mallipattu S.K. (2019). Therapeutic Inhibition of VEGF Signaling and Associated Nephrotoxicities. J. Am. Soc. Nephrol..

[22] Alikhan M.A., Jones C.V., Williams T.M., Beckhouse A.G., Fletcher A.L., Kett M.M., Sakkal S. (2011). Colony-stimulating factor-1 promotes kidney growth and repair via alteration of macrophage responses. Am. J. Pathol..

[23] Uchio K., Sawada K., Manabe N. (2009). Expression of macrophage metalloelastase (MMP-12) in podocytes of hereditary nephrotic mice (ICGN strain). J. Vet. Med. Sci..

[24] Lee J.M., Ko Y., Lee C.H., Jeon N., Lee K.H., Oh J., Kronbichler A., Saleem M.A., Lim B.J., Shin J.I. (2021). The Effect of Interleukin-4 and Dexamethasone on RNA-Seq-Based Transcriptomic Profiling of Human Podocytes: A Potential Role in Minimal Change Nephrotic Syndrome. J. Clin. Med..

[25] Kang J., Bai K.M., Wang B.L., Yao Z., Pang X.W., Chen W.F. (1994). Increased production of interleukin 4 in children with simple idiopathic nephrotic syndrome. Chin. Med. J. (Engl).

[26] Riedel J.H., Paust H.J., Krohn S., Turner J.E., Kluger M.A., Steinmetz O.M., Krebs C.F., Stahl R.A., Panzer U. (2016). IL-17F Promotes Tissue Injury in Autoimmune Kidney Diseases. J. Am. Soc. Nephrol..

[27] Chen H., Zhang Z., Zhou L., Cai T., Liu B., Wang L., Yang J. (2022). Identification of CCL19 as a Novel Immune-Related Biomarker in Diabetic Nephropathy. Front. Genet..

[28] Komatsuda A., Wakui H., Iwamoto K., Harada M., Okumoto Y., Sawada K. (2008). Gene expression profiling of peripheral blood mononuclear cells from patients with minimal change nephrotic syndrome by cDNA microarrays. Am. J. Nephrol..

[29] Tian G., Lu J., Guo H., Liu Q., Wang H. (2015). Protective effect of Flt3L on organ structure during advanced multiorgan dysfunction syndrome in mice. Mol. Med. Rep..

[30] Hodson E.M., Sinha A., Cooper T.E. (2022). Interventions for focal segmental glomerulosclerosis in adults. Cochrane Database Syst. Rev..

