# Supplementary material for: The Role of Cytokines and Chemokines as Biomarkers of Disease Activity in Idiopathic Nephrotic Syndrome in Children
Source: Curr Issues Mol Biol. 2025 Jan 25;47(2):77. doi: 10.3390/cimb47020077 (PMC11854277; doi:10.3390/cimb47020077)
Supplement: Supplementary file 1 [file cimb-47-00077-s001.zip › cimb-3413479-supplementary.pdf]

Supplementary material - tables and graphs

Table S1 a. Comparison of statistical significance of concentration differences of cytokines and chemokines in plasma samples of patients at disease onset or relapse compared to those in remission achieved with corticosteroid (CS) treatment

cytokine / chemokine	p-value	p-value wilcoxon	Number of paired samples
CSF1	0.0010033	0.0017090	13
MMP12	0.0364303	0.0327148	13
FLT3LG	0.0501476	0.0397949	13
IL4	0.1103888	0.0419922	11
CSF3	0.0868512	0.0522461	12
CCL7	0.1967962	0.0573730	13
CCL19	0.0423872	0.0681152	13
CCL11	0.1307934	0.0942383	13
CXCL12	0.1904329	0.1464844	13
IL13	0.3914093	0.1484375	8
IL6	0.1695239	0.1762695	12
TNFSF10	0.2248418	0.1909180	13
IFNG	0.6348540	0.1909180	13
CXCL11	0.2328878	0.2031250	9
IL17C	0.2441033	0.2163086	13
CSF2	0.2652395	0.2438965	13
VEGFA	0.1840756	0.2438965	13
TSLP	0.3516849	0.2500000	3
TNF	0.4809276	0.2734375	13
CCL3	0.2182816	0.3393555	12
IL17A	0.3144213	0.3393555	12
IL33	0.2008420	0.3750000	7
CCL2	0.3463362	0.4143066	13
LTA	0.2365707	0.4548340	13
IL27	0.4959842	0.5417480	13
HGF	0.9385564	0.5417480	13
CCL4	0.2158542	0.5693359	12

cytokine / chemokine	p-value	p-value wilcoxon	Number of paired samples
IL7	0.3813194	0.5878906	13
CXCL10	0.3995374	0.5878906	13
IL15	0.7369948	0.6354980	13
IL2	0.7445293	0.6772461	12
IL1B	0.7225597	0.6848145	13
OSM	0.6838425	0.6848145	13
MMP1	0.4382714	0.7353516	13
OLR1	0.9208165	0.7868652	13
IL17F	0.3991206	0.7868652	13
IL18	0.6005392	0.7868652	13
CXCL9	0.4010400	0.8925781	13
TGFA	0.7192293	0.8925781	13
TNFSF12	0.8160226	0.8925781	13
CCL13	0.4870152	0.8925781	13
IL10	0.4197316	0.8925781	13
CCL8	0.6864434	0.9460449	13
EGF	0.9581424	0.9460449	13
CXCL8	0.7021803	1.0000000	13

Table S1 b. Results of laboratory measurements (average, minimal, maximal values) of concentrations (in pg/ml) of various cytokines and chemokines in plasma samples of patients at disease onset or relapse (group 1 – G1) and in those in remission achieved with CS treatment (group 2 – G2) and statistical significance of concentration differences among the two groups.

cytokine / chemokine	G1 average	G1 [min,max]	G2 average	G2 [min,max]	p-value	p-value wilcoxon	Number of paired samples
CSF1	17914680.23	[13500147,22343194]	13484454.92	[9643395,16363254]	0.0010033	0.0017090	13
MMP12	28607545.92	[6436080,58715052]	15874352.54	[3696656,43664751]	0.0364303	0.0327148	13
FLT3LG	8445538.46	[2788728,18855889]	5741948.69	[1951467,9135271]	0.0501476	0.0397949	13
IL4	0.0392	[0.008,0.1632]	0.0102	[0.0072,0.0175]	0.1103888	0.0419922	11
CSF3	10712159.67	[4541409,24322226]	7037298.42	[3503581,18314568]	0.0868512	0.0522461	12
CCL7	601980.46	[120166,4025539]	194368.75	[0.7195,465334]	0.1967962	0.0573730	13

cytokine / chemokine	G1 average	G1 [min,max]	G2 average	G2 [min,max]	p-value	p-value wilcoxon	Number of paired samples
CCL19	10939782.31	[2311752,31713696]	6144620.54	[912614,13383068]	0.0423872	0.0681152	13
CCL11	29137349.77	[21574141,43779310]	34443222.77	[10561458,52522863]	0.1307934	0.0942383	13
CXCL12	12658870.15	[9590416,15964493]	11028584.38	[3984800,18405977]	0.1904329	0.1464844	13
IL13	76335.93	[0.0022,455694]	18292.32	[0.0051,146338]	0.3914093	0.1484375	8
IL6	381966.32	[0.818,870221]	186369.35	[0.1152,947934]	0.1695239	0.1762695	12
TNFSF10	55467624.62	[28640761,89242329]	47114393.23	[24446446,79944345]	0.2248418	0.1909180	13
IFNG	33852.67	[0.1278,331788]	17702.49	[0.0325,230130]	0.6348540	0.1909180	13
CXCL11	74682656.33	[29097149,148400026]	52106976.89	[20478155,127761330]	0.2328878	0.2031250	9
IL17C	5583129.77	[1588835,16266497]	4003677.46	[995129,11357197]	0.2441033	0.2163086	13
CSF2	0.2416	[0.0655,0.5998]	0.1654	[0.0186,0.5502]	0.2652395	0.2438965	13
VEGFA	53534612.62	[14340464,153340869]	34890548	[17217950,88650132]	0.1840756	0.2438965	13
TSLP	0.1129	[0.0284,0.2716]	0.0125	[0.0044,0.0204]	0.3516849	0.2500000	3
TNF	11030493.15	[1186160,57114694]	6089851.38	[524372,45583698]	0.4809276	0.2734375	13
CCL3	14396536.33	[306682,95556554]	2411728.83	[269530,14928773]	0.2182816	0.3393555	12
IL17A	153656.9	[0.0108,398454]	89289.35	[0.0329,330979]	0.3144213	0.3393555	12
IL33	0.1058	[0.0144,0.2325]	0.0616	[0.0315,0.0959]	0.2008420	0.3750000	7
CCL2	54024919.31	[20435657,185374295]	40284047.38	[10062980,75754591]	0.3463362	0.4143066	13
LTA	1362806.69	[496684,3378481]	990039.38	[270766,2509107]	0.2365707	0.4548340	13
IL27	788946.46	[0.9915,2981419]	1035071.38	[127791,3800994]	0.4959842	0.5417480	13
HGF	24881971.15	[8321877,92999075]	24420734.15	[5823270,45443052]	0.9385564	0.5417480	13
CCL4	48287898.83	[3226756,258750150]	13651738	[4196127,55305670]	0.2158542	0.5693359	12
IL7	64881.07	[0.482,212075]	118572.06	[0.489,804032]	0.3813194	0.5878906	13
CXCL10	12623374.38	[2989336,75998289]	7760392.08	[1363335,26564907]	0.3995374	0.5878906	13
IL15	1453812.31	[568572,2939418]	1389722.38	[700485,2383005]	0.7369948	0.6354980	13
IL2	0.0278	[0.0024,0.1084]	0.0346	[0.0034,0.1993]	0.7445293	0.6772461	12
IL1B	160892.38	[0.0501,1111677]	108030.3	[0.0526,1258258]	0.7225597	0.6848145	13
OSM	2313980.69	[162562,13203573]	1929911.46	[309628,4714691]	0.6838425	0.6848145	13
MMP1	78559749.92	[17967288,265674836]	60548382.62	[15260718,158394394]	0.4382714	0.7353516	13
OLR1	55958418.69	[10149197,99012758]	57065214.31	[23771150,101170843]	0.9208165	0.7868652	13
IL17F	70317.86	[0.3335,487266]	144152.28	[0.1815,640824]	0.3991206	0.7868652	13
IL18	37818514.54	[22062388,63881973]	34836187	[18548732,56017222]	0.6005392	0.7868652	13
CXCL9	7569628.08	[1981714,16590777]	12307788.69	[1597166,69717318]	0.4010400	0.8925781	13

cytokine / chemokine	G1 average	G1 [min,max]	G2 average	G2 [min,max]	p-value	p-value wilcoxon	Number of paired samples
TGFA	3689447.31	[619115,10898998]	4217569.77	[449085,16091934]	0.7192293	0.8925781	13
TNFSF12	43872218.77	[18329076,63551828]	42993452	[32183523,75475058]	0.8160226	0.8925781	13
CCL13	28408096.77	[6081243,83490547]	22152028.08	[9981482,53454440]	0.4870152	0.8925781	13
IL10	2476229.69	[536220,15791545]	1670207.08	[216149,3892897]	0.4197316	0.8925781	13
CCL8	2824077.38	[760140,5218733]	2627300.15	[933231,5707848]	0.6864434	0.9460449	13
EGF	16993839.23	[1754581,41520827]	16692711.69	[4342466,49276826]	0.9581424	0.9460449	13
CXCL8	13176706.31	[733172,83261504]	20514295.15	[835746,214685851]	0.7021803	1.0000000	13

Graph S1. Presentation of numbers of paired samples ("stevilo parov") according to investigated cytokine / chemokine ("spremenljivka")

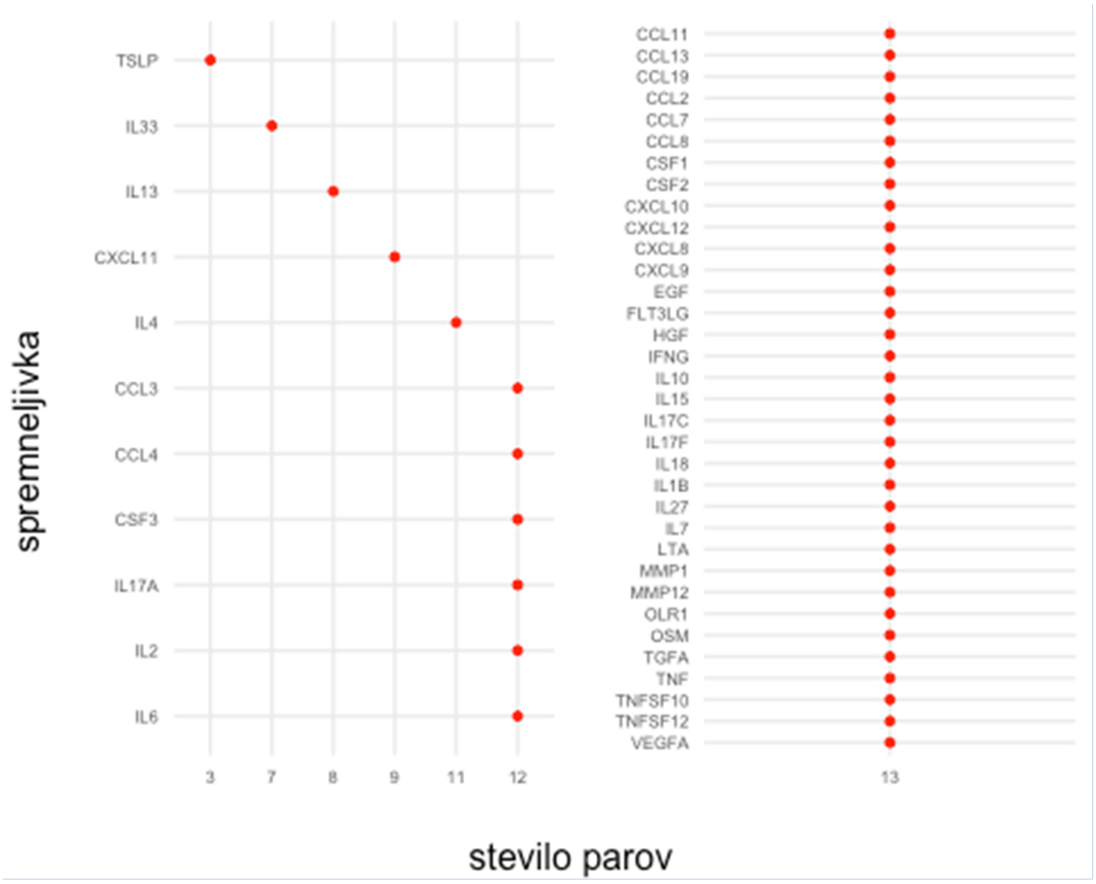

Table S2 a. Comparison of statistical significance of concentration differences of cytokines and chemokines in plasma samples of patients at disease onset or relapse compared to those in remission after conclusion of CS treatment

cytokine / chemokine	p-value	p-value wilcoxon	Number of paired samples
CSF1	0.0030420	0.0156250	8
IL17F	0.0676425	0.0390625	8
CSF2	0.1123186	0.0781250	8
TGFA	0.1462047	0.1093750	8
IL6	0.1002051	0.1093750	7
CCL13	0.0904795	0.1093750	8
OLR1	0.1915458	0.1484375	8
CCL11	0.1046880	0.1484375	8
IL15	0.1721032	0.1484375	8
VEGFA	0.1266494	0.1484375	8
IL17C	0.1031866	0.1484375	8
CCL19	0.3226174	0.1953125	8
OSM	0.2465427	0.1953125	8
CSF3	0.2397360	0.2187500	7
IL13	0.3559164	0.2968750	7
CXCL9	0.4148639	0.3125000	8
TNF	0.1702484	0.3125000	8
IL2	0.2219055	0.3828125	8
IL1B	0.1415515	0.3828125	8
HGF	0.2687245	0.3828125	8
CXCL10	0.6604782	0.3828125	8
CCL3	0.2055868	0.3828125	8
CXCL8	0.1913742	0.3828125	8
CCL7	0.3089838	0.3828125	8
IL27	0.4351760	0.4609375	8
CXCL12	0.9999066	0.5468750	8
IL7	0.8079866	0.5468750	8
EGF	0.5684036	0.5468750	8
CXCL11	0.4941166	0.5625000	6
CCL8	0.4435185	0.6406250	8
MMP12	0.2709125	0.6406250	8

cytokine / chemokine	p-value	p-value wilcoxon	Number of paired samples
MMP1	0.3545121	0.6406250	8
IL4	0.7489098	0.7421875	8
CCL2	0.8316792	0.7421875	8
IL33	0.5266601	0.8125000	5
TNFSF12	0.4572986	0.8437500	8
FLT3LG	0.7270675	0.8437500	8
IFNG	0.9626523	0.8437500	8
IL17A	0.9501522	0.8437500	8
CCL4	0.1926673	0.8437500	8
IL18	0.5766346	0.9453125	8
TNFSF10	0.8664835	0.9453125	8
IL10	0.3544383	0.9453125	8
LTA	0.9088178	1.0000000	8

Table S2 b. Results of laboratory measurements (average, minimal, maximal values) of concentrations (in pg/ml) of various cytokines and chemokines in plasma samples of patients at disease onset or relapse (group 1 – G1) and in those in remission after conclusion of CS treatment (group 3 – G3) and statistical significance of concentration differences among the two groups.

cytokine/ chemokine	G1 average	G1 [min,max]	G3 average	G3 [min,max]	p-value	p-value wilcoxon	Number of paired samples
CSF1	**18456847.62**	[13500147,22343194]	13776127.75	[11727921,15216580]	0.0030420	0.0156250	8
IL17F	21062.09	[0.3415,168493]	85123.07	[0.1962,209438]	0.0676425	0.0390625	8
CSF2	0.2621	[0.0655,0.5998]	0.131	[0.072,0.3468]	0.1123186	0.0781250	8
TGFA	3403870	[619115,10898998]	1429823.62	[1038268,2699243]	0.1462047	0.1093750	8
IL6	441735.29	[145548,750131]	260800	[123915,481676]	0.1002051	0.1093750	7
CCL13	19338434.88	[6081243,41561871]	32143810.25	[7983753,54187328]	0.0904795	0.1093750	8
OLR1	56199485.62	[10149197,99012758]	37090598.88	[10581147,55664920]	0.1915458	0.1484375	8
CCL11	29009675.38	[21574141,43779310]	22226906.5	[7287531,33657776]	0.1046880	0.1484375	8
IL15	1463305.25	[568572,2939418]	1064514.75	[705456,1470624]	0.1721032	0.1484375	8
VEGFA	57012623	[18654802,153340869]	28006300.62	[17137827,68159777]	0.1266494	0.1484375	8
IL17C	6255790	[1588835,16266497]	2378501.75	[1159049,6991050]	0.1031866	0.1484375	8
CCL19	13291988.12	[5933893,31713696]	17541202.12	[8872624,29817318]	0.3226174	0.1953125	8
OSM	2808802.88	[162562,13203573]	808963.5	[396842,1770045]	0.2465427	0.1953125	8

cytokine/ chemokine	G1 average	G1 [min,max]	G3 average	G3 [min,max]	p-value	p-value wilcoxon	Number of paired samples
CSF3	13205296.29	[6850405,24322226]	17251966.14	[6404327,25171476]	0.2397360	0.2187500	7
IL13	65099.48	[0.0016,455694]	17281.08	[0.0326,120966]	0.3559164	0.2968750	7
CXCL9	8655621.62	[2495222,16590777]	10455109.75	[3019834,24883095]	0.4148639	0.3125000	8
TNF	16050364.12	[1186160,57114694]	3487291	[966544,13205673]	0.1702484	0.3125000	8
IL2	0.0308	[0.0034,0.1084]	0.0152	[0.0072,0.0274]	0.2219055	0.3828125	8
IL1B	245742.01	[0.128,1111677]	0.2185	[0.0499,0.4171]	0.1415515	0.3828125	8
HGF	27410329.88	[8321877,92999075]	14814811.75	[10961853,33507934]	0.2687245	0.3828125	8
CXCL10	16253556.12	[3591961,75998289]	12023612.12	[3764268,23407805]	0.6604782	0.3828125	8
CCL3	20291235.25	[467293,95556554]	1833963.12	[308736,9414915]	0.2055868	0.3828125	8
CXCL8	17815137.75	[733172,83261504]	1850041.12	[567500,6889234]	0.1913742	0.3828125	8
CCL7	758402.75	[120166,4025539]	228147.35	[0.8229,421314]	0.3089838	0.3828125	8
IL27	640152	[139422,1147853]	527723.5	[111487,1040384]	0.4351760	0.4609375	8
CXCL12	11794722.38	[9590416,14313210]	11794885.62	[8139017,18657168]	0.9999066	0.5468750	8
IL7	63514.33	[0.482,191966]	80910.96	[0.2678,429421]	0.8079866	0.5468750	8
EGF	15164697.88	[1766823,41520827]	10518230.62	[1526211,43409477]	0.5684036	0.5468750	8
CXCL11	92501191	[47836198,148400026]	70288394	[3527773,190654379]	0.4941166	0.5625000	6
CCL8	2666145	[760140,5218733]	3145581.5	[887209,9002006]	0.4435185	0.6406250	8
MMP12	31615696.25	[13180059,58715052]	42074979.12	[15489894,74369292]	0.2709125	0.6406250	8
MMP1	72810269.5	[17967288,265674836]	44080940.12	[14487935,79707439]	0.3545121	0.6406250	8
IL4	0.033	[0.008,0.1632]	0.0256	[0.0062,0.0701]	0.7489098	0.7421875	8
CCL2	58041583.75	[20435657,185374295]	53390797.12	[25524533,114695661]	0.8316792	0.7421875	8
IL33	0.1255	[0.0144,0.2325]	0.1687	[0.0211,0.3116]	0.5266601	0.8125000	5
TNFSF12	43985258.5	[18329076,63551828]	56774637.75	[33685713,157109541]	0.4572986	0.8437500	8
FLT3LG	8239748.38	[5356144,10987766]	8500369.12	[7008877,10384425]	0.7270675	0.8437500	8
IFNG	13536.95	[0.1732,108293]	12578.51	[0.1171,100626]	0.9626523	0.8437500	8
IL17A	182985.47	[0.0108,398454]	188324.92	[0.315,722534]	0.9501522	0.8437500	8
CCL4	64820480.12	[5500495,258750150]	10506357	[4165148,20564167]	0.1926673	0.8437500	8
IL18	38706239.75	[22062388,61594539]	35349287.5	[22177784,60050877]	0.5766346	0.9453125	8
TNFSF10	53436587	[28640761,83109230]	54502842.25	[36268109,70791378]	0.8664835	0.9453125	8
IL10	3500171.75	[743017,15791545]	1650755.75	[433096,3043415]	0.3544383	0.9453125	8
LTA	1598568.62	[496684,3378481]	1634537.38	[459801,2474056]	0.9088178	1.0000000	8

Graph S2. Presentation of numbers of paired samples ("stevilo parov") according to investigated cytokine / chemokine ("spremenljivka")

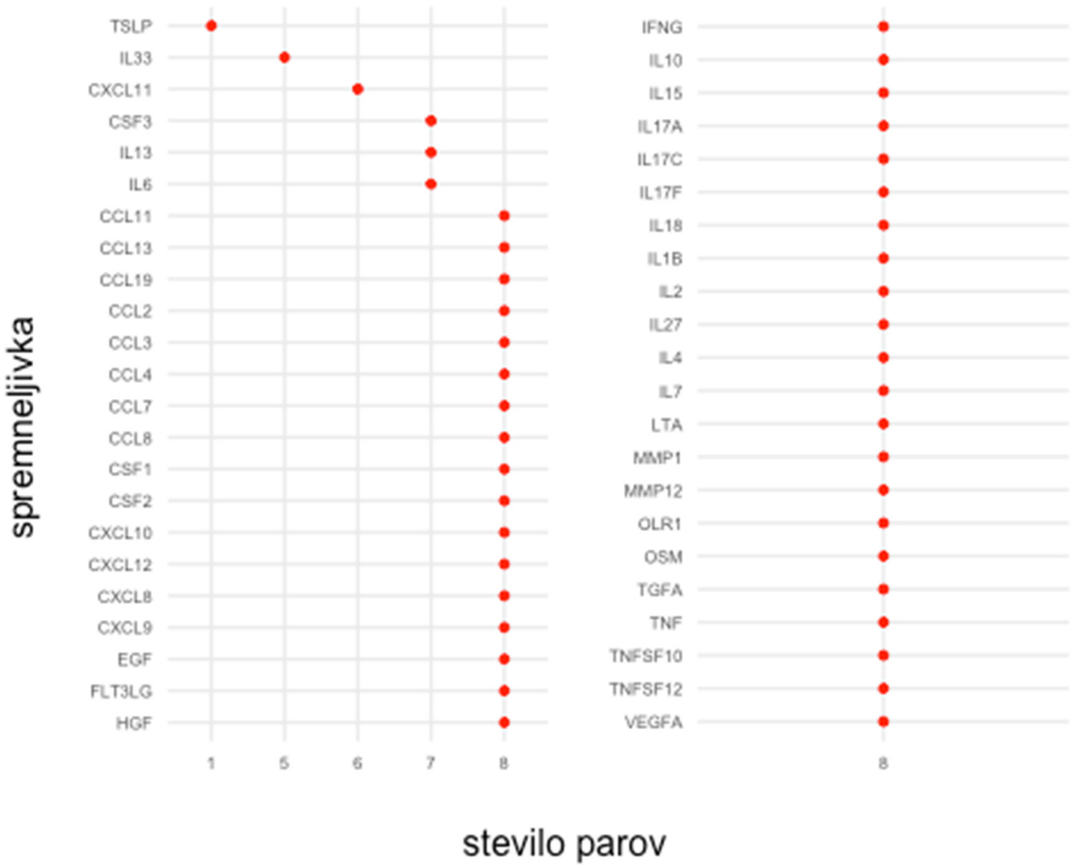

Table S3 a. Comparison of statistical significance of concentration differences of cytokines and chemokines in plasma samples of patients at remission achieved with CS treatment compared to those in remission after conclusion of CS treatment

cytokine / chemokine	p-value	p-value wilcoxon	Number of paired samples
CCL19	0.0153748	0.0156250	8
MMP12	0.0323816	0.0390625	8
CCL13	0.0419881	0.0546875	8
CCL11	0.0688743	0.0781250	8
IL4	0.0804537	0.1093750	8
FLT3LG	0.0780905	0.1093750	8
IL17C	0.2141270	0.1093750	8
TGFA	0.1492200	0.1484375	8
HGF	0.1263166	0.1484375	8
LTA	0.1463113	0.1484375	8
CSF3	0.1475792	0.1953125	8
IL27	0.1774670	0.2500000	8
TNF	0.2482235	0.2500000	8
IL15	0.3611428	0.2500000	8
OSM	0.1760196	0.2500000	8
CXCL12	0.5746958	0.3125000	8
IL2	0.2619912	0.3125000	8
VEGFA	0.5641915	0.3125000	8
EGF	0.5904250	0.3828125	8
CXCL10	0.3890625	0.4609375	8
CXCL8	0.6106466	0.4609375	8
CCL2	0.3484916	0.4609375	8
IL17A	0.4667487	0.4687500	7
IL6	0.3906145	0.5468750	8
IL7	0.5396545	0.5468750	8
IFNG	0.6387646	0.5468750	8
IL10	0.6279878	0.5468750	8
CSF2	0.5721295	0.6406250	8
CCL7	0.4679086	0.6406250	8
IL13	0.9075254	0.6875000	6
IL1B	0.4463905	0.7421875	8

cytokine / chemokine	p-value	p-value wilcoxon	Number of paired samples
TNFSF10	0.6850901	0.7421875	8
MMP1	0.4458630	0.7421875	8
TSLP	0.4226498	0.7500000	3
OLR1	0.5217201	0.8437500	8
CXCL9	0.5972675	0.8437500	8
TNFSF12	0.5583301	0.8437500	8
CSF1	0.8565730	0.9453125	8
CCL4	0.8036082	0.9453125	8
CCL8	0.4350852	0.9453125	8
IL17F	0.9141772	0.9453125	8
IL33	0.6633897	1.0000000	2
IL18	0.7990468	1.0000000	8
CCL3	0.8488266	1.0000000	8
CXCL11	0.8752788	1.0000000	5

Table S3 b. Results of laboratory measurements (average, minimal, maximal values) of concentrations (in pg/ml) of various cytokines and chemokines in plasma samples of patients at remission achieved with CS treatment (group 2 – G2) and in those in remission after conclusion of CS treatment (group 3 – G3) and statistical significance of concentration differences among the two groups.

cytokine/ chemokine	G2 average	G2 [min,max]	G3 average	G3 [min,max]	p-value	p-value wilcoxon	Number of paired samples
CCL19	7743258.5	[2344128,13383068]	**17541202.12**	[8872624,29817318]	0.0153748	0.0156250	8
MMP12	18486897.25	[3696656,43664751]	**42074979.12**	[15489894,74369292]	0.0323816	0.0390625	8
CCL13	16545436	[9981482,26466910]	**32143810.25**	[7983753,54187328]	0.0419881	0.0546875	8
CCL11	32520183.75	[10561458,48772022]	22226906.5	[7287531,33657776]	0.0688743	0.0781250	8
IL4	0.0097	[0.0072,0.0175]	0.0256	[0.0062,0.0701]	0.0804537	0.1093750	8
FLT3LG	6642175.5	[4106626,9135271]	8500369.12	[7008877,10384425]	0.0780905	0.1093750	8
IL17C	4512594.75	[1591074,11357197]	2378501.75	[1159049,6991050]	0.2141270	0.1093750	8
TGFA	2436083.88	[449085,5010303]	1429823.62	[1038268,2699243]	0.1492200	0.1484375	8
HGF	21745193	[5823270,38204448]	14814811.75	[10961853,33507934]	0.1263166	0.1484375	8
LTA	1167830.25	[270766,2509107]	1634537.38	[459801,2474056]	0.1463113	0.1484375	8
CSF3	8757473.38	[4016238,18314568]	15673997.88	[4628220,25171476]	0.1475792	0.1953125	8
IL27	1264940.62	[344966,3800994]	527723.5	[111487,1040384]	0.1774670	0.2500000	8
TNF	2017743	[524372,4186207]	3487291	[966544,13205673]	0.2482235	0.2500000	8

cytokine/ chemokine	G2 average	G2 [min,max]	G3 average	G3 [min,max]	p-value	p-value wilcoxon	Number of paired samples
IL15	1268276.25	[700485,2059335]	1064514.75	[705456,1470624]	0.3611428	0.2500000	8
OSM	1347506	[309628,2904364]	808963.5	[396842,1770045]	0.1760196	0.2500000	8
CXCL12	10704238.88	[3984800,18405977]	11794885.62	[8139017,18657168]	0.5746958	0.3125000	8
IL2	0.0237	[0.0034,0.0772]	0.0152	[0.0072,0.0274]	0.2619912	0.3125000	8
VEGFA	32616201	[18002705,45109632]	28006300.62	[17137827,68159777]	0.5641915	0.3125000	8
EGF	13986474.25	[6226272,39700988]	10518230.62	[1526211,43409477]	0.5904250	0.3828125	8
CXCL10	9317690.75	[1735025,26564907]	12023612.12	[3764268,23407805]	0.3890625	0.4609375	8
CXCL8	2350808.25	[835746,7698297]	1850041.12	[567500,6889234]	0.6106466	0.4609375	8
CCL2	41784356.5	[26328273,75754591]	53390797.12	[25524533,114695661]	0.3484916	0.4609375	8
IL17A	92292.17	[0.1812,330979]	182096.77	[0.315,722534]	0.4667487	0.4687500	7
IL6	147586.69	[0.5516,429328]	228200.11	[0.9137,481676]	0.3906145	0.5468750	8
IL7	161520.85	[0.489,804032]	80910.96	[0.2678,429421]	0.5396545	0.5468750	8
IFNG	28766.44	[0.0325,230130]	12578.51	[0.1171,100626]	0.6387646	0.5468750	8
IL10	1992614	[216149,3892897]	1650755.75	[433096,3043415]	0.6279878	0.5468750	8
CSF2	0.172	[0.0416,0.4348]	0.131	[0.072,0.3468]	0.5721295	0.6406250	8
CCL7	189609.71	[0.7195,465334]	228147.35	[0.8229,421314]	0.4679086	0.6406250	8
IL13	24389.78	[0.0089,146338]	20161.33	[0.098,120966]	0.9075254	0.6875000	6
IL1B	0.3028	[0.101,0.8181]	0.2185	[0.0499,0.4171]	0.4463905	0.7421875	8
TNFSF10	52019592.88	[38869447,79944345]	54502842.25	[36268109,70791378]	0.6850901	0.7421875	8
MMP1	59324942.88	[15261308,158394394]	44080940.12	[14487935,79707439]	0.4458630	0.7421875	8
TSLP	37092.01	[0.0056,111276]	0.0195	[0.0123,0.0295]	0.4226498	0.7500000	3
OLR1	43974603.5	[23771150,96461301]	37090598.88	[10581147,55664920]	0.5217201	0.8437500	8
CXCL9	14858829.25	[1597166,69717318]	10455109.75	[3019834,24883095]	0.5972675	0.8437500	8
TNFSF12	46641326.88	[35771376,75475058]	56774637.75	[33685713,157109541]	0.5583301	0.8437500	8
CSF1	13940288.12	[11478927,16363254]	13776127.75	[11727921,15216580]	0.8565730	0.9453125	8
CCL4	11489150.12	[4295824,20851826]	10506357	[4165148,20564167]	0.8036082	0.9453125	8
CCL8	2474032.62	[933231,5707848]	3145581.5	[887209,9002006]	0.4350852	0.9453125	8
IL17F	94432.46	[0.1815,640824]	85123.07	[0.1962,209438]	0.9141772	0.9453125	8
IL33	0.0737	[0.0524,0.0951]	0.1456	[0.0439,0.2473]	0.6633897	1.0000000	2
IL18	36886293	[25593357,56017222]	35349287.5	[22177784,60050877]	0.7990468	1.0000000	8
CCL3	1607146.5	[499555,5858414]	1833963.12	[308736,9414915]	0.8488266	1.0000000	8
CXCL11	42271766	[7101031,82008021]	46215197	[3527773,114621231]	0.8752788	1.0000000	5

Graph S3. Presentation of numbers of paired samples ("stevilo parov") according to investigated cytokine / chemokine ("spremenljivka")

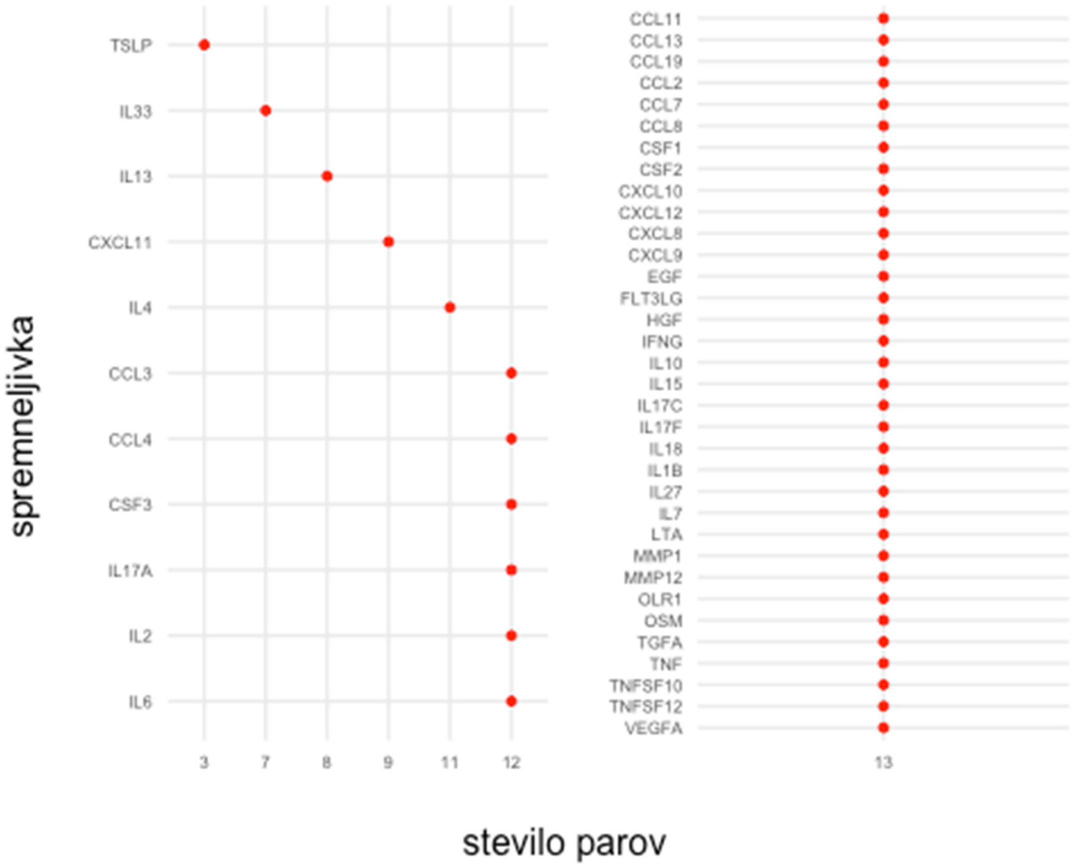

Table S4. Statistically significant concentration differences of cytokines and chemokines in plasma samples of patients at disease onset or relapse (group 1 – G1) compared to those in remission achieved with CS treatment (group 2 – G2) and to those in remission after conclusion of CS treatment (group 3 – G3).

Comparison between groups (G)	cytokine/ chemokine	G1 average	G1 [min,max]	G2 average	G2 [min,max]	p-value	p-value wilcoxon	Number of paired samples
1-2	CSF1	17914680.23	[13500147,22343194]	13484454.92	[9643395,16363254]	0.0010	0.0017	13
1-2	MMP12	28607545.92	[6436080,58715052]	15874352.54	[3696656,43664751]	0.0364	0.0327	13
1-2	FLT3LG	8445538.46	[2788728,18855889]	5741948.69	[1951467,9135271]	0.0501	0.0398	13
1-2	IL4	0.0392	[0.008,0.1632]	0.0102	[0.0072,0.0175]	0.1104	0.0420	11
1-2	CCL19	10939782.31	[2311752,31713696]	6144620.54	[912614,13383068]	0.0424	0.0681	13
		**G1 average**	**G1 [min,max]**	**G3 average**	**G3 [min,max]**			
1-3	CSF1	18456847.62	[13500147,22343194]	13776127.75	[11727921,15216580]	0.0030	0.0156	8
1-3	IL17F	21062.09	[0.3415,168493]	85123.07	[0.1962,209438]	0.0676	0.0391	8
		**G2 average**	**G2 [min,max]**	**G3 average**	**G3 [min,max]**			
2-3	CCL19	7743258.5	[2344128,13383068]	17541202.12	[8872624,29817318]	0.0154	0.0156	8
2-3	MMP12	18486897.25	[3696656,43664751]	42074979.12	[15489894,74369292]	0.0324	0.0391	8
2-3	CCL13	16545436	[9981482,26466910]	32143810.25	[7983753,54187328]	0.0420	0.0547	8
